# Machine Learning and Weighted Gene Coexpression Network–Based Identification of Biomarkers Predicting Immune Profiling and Drug Resistance in Lung Adenocarcinoma

**DOI:** 10.1155/ijog/9923294

**Published:** 2025-03-22

**Authors:** Tian Zhang, Han Zhou

**Affiliations:** Pharmacy Department, Xiangxi Autonomous Prefecture People's Hospital, Jishou, China

**Keywords:** drug sensitivity, immune infiltration, lung adenocarcinoma, prognostic models, WGCNA

## Abstract

**Background:** The prognosis for lung adenocarcinoma (LUAD) is poor, and the recurrence rate is high. Thus, to evaluate patients' prognoses and direct therapy choices, new prognostic markers are desperately needed.

**Methods:** First, gene modules associated with LUAD were identified by weighted gene coexpression network analysis (WGCNA) analysis. The expression profiles obtained were intersected with the differential expressed genes taken between LUAD samples and paracancerous samples. Afterward, stepwise regression analysis and the LASSO were used to compress the genes further, and a risk model was created. Furthermore, a nomogram based on risk scores and clinical features was created to validate the model. After that, the distinctions between the pertinent biological processes and signaling pathways among the various subgroups were investigated. Additionally, drug sensitivity testing, immunotherapy, immune infiltration analysis, and enrichment analysis were carried out. Finally, the biological role of *ANLN* in LUAD was explored by qPCR, cell scratch assay, and transwell.

**Results:** A total of 257 intersected genes were obtained by taking the intersection of the differential genes between 2866 LUAD samples and paraneoplastic samples with the module genes after we screened two particular modules that had the strongest link with LUAD by WGCNA. *ANLN*, *CASS4*, and *NMUR1* were found to be distinctive genes for the development of risk models after the intersecting genes were screened to find 176 genes linked to the prognosis for LUAD. Based on risk assessments, high- and low-risk groups of LUAD patients were divided. Low-risk patients exhibited a significantly higher overall survival (OS) than those in the high-risk group. Expression of model genes correlates with infiltration of the vast majority of immune cells. Significant differences in the biological pathways, immune microenvironment, and abundance of immune cell infiltration were found between the two groups. The drug sensitivity study showed that patients in the high-risk group had higher IC_50_ values for BMS-754807_2171 and Doramapimod_10424. Finally, in vitro experiments demonstrated that knocking down *ANLN* noticeably inhibited the viability, migration, and invasion of A549 cells.

**Conclusion:** This study may provide a theoretical reference for future exploration of potential diagnostic and prognostic biomarkers for LUAD.

## 1. Introduction

Statistics showed that lung cancer is a major cause leading to cancer-related fatalities [1–3]. As the most common histological subtype of lung cancer, lung adenocarcinoma (LUAD) accounts for 40%–50% of lung cancer cases [4, 5]. The prognosis for LUAD is dismal, and treatment is difficult. Patients with early-stage LUAD typically receive surgical resection, chemotherapy, and radiation therapy, according to early research [6, 7]. Unfortunately, the prognosis for many patients is dismal, since after 5 years, recurrence rates range from 20% in Stage I patients to 50% in Stage III patients [8]. Significant progress has been made for LUAD, despite the fact that researchers are constantly looking for novel approaches and treatments, such as surgical resection coupled with chemotherapy or combination therapies like radiotherapy and immunotherapy. However, the medicines' numerous dangers and side effects make it difficult to determine how they will affect survival and long-term efficacy, which continues to be a barrier to cancer treatment. For instance, surgery is the usual course of treatment for localized LUAD; yet, the overall 5-year survival chance is still less than 50%. According to statistics, patients who experience distant metastases have a 7% 5-year survival rate [9]. Furthermore, patients' treatment landscapes have been altered by pharmaceutical immunotherapy targeting the PD-1/PD-L1 axis; nonetheless, most patients suffer primary resistance, with objective response rates to immune checkpoint blockade (ICB) monotherapy ranging from 10% to 30% [10, 11]. While radiotherapy is the usual course of treatment for individuals with lung cancer, individual patients respond differently to it, particularly if they have LUAD. LUAD patients are more radiation-resistant than other patient populations. Radiation resistance can result in tumor recurrence and metastasis and is a primary cause of therapy failure in LUAD [12]. Because of metastasis and recurrence, the prognosis for LUAD patients is still dismal despite advancements in cancer treatment. Thus, to evaluate patients' prognoses and direct therapy choices, new prognostic markers are desperately needed.

Microarray data that are currently available to identify hub genes, interaction networks, and pathways in LUAD thanks to advancements in bioinformatics. WGCNA is a very methodical bioinformatics methodology, notwithstanding the drawbacks of classical experiments [13]. It gives researchers the chance to conduct higher resolution analyses in order to more accurately pinpoint the key functioning genes that could yield a stronger biological signature for phenotypic characterization and, consequently, more appropriate biomarker candidates for later research [14]. In addition to combining various informatics techniques, such as Kyoto Encyclopedia of Genes and Genomes (KEGG) enrichment analysis and gene set enrichment analysis, to screen genes highly associated with diseases integrated into multiple modules to reveal underlying molecular mechanisms, WGCNA can be used to construct expression profiles of mRNAs in diseases [15]. Thus, this study set out to identify new biomarkers, genes, or putative pathways linked to LUAD.

In order to identify the critical genes linked to prognosis, we conducted a WGCNA of the mRNA expression profiles in LUAD. We then took the intersection of the expression profiles obtained with the data of differential expression profiles between LUAD samples and paracancerous samples. Next, a risk score system was developed for grouping high- and low-risk patients. We also examined the relationship between immune infiltration and hub genes and investigated the variations in associated biological processes and signaling pathways among various subpopulations. Lastly, an analysis was done on the relationship between the risk model and immunotherapy and medication sensitivity. Overall, by combining multiple popular bioinformatics algorithms for LUAD, our study offers a new way to predict prognosis-related features for patients, drug sensitivity, and treatment effectiveness of immune checkpoint inhibitors (ICIs). This approach may offer new insights into prognostic assessment and therapeutic strategies for LUAD.

## 2. Methods

### 2.1. Data Acquisition

The UCSC Xena database (https://xena.ucsc.edu/) provided us with the count with reads per million mapped (FPKM) format of the LUAD dataset as well as all follow-up data. We kept the samples with missing clinical data, with the exception of those without surviving follow-up data. A total of 527 samples were acquired, comprising 474 cancer samples and 53 paraneoplastic samples, from adenocarcinoma 01A cancer samples and adenoma 11A paraneoplastic samples. We obtained the GSE31210 and GSE50081 cohorts from the Gene Expression Omnibus (GEO, https://www.ncbi.nlm.nih.gov/geo/) database, excluding the LUAD patients with survival times < 30 days. This resulted in 226 and 127 samples, respectively, serving as the validation set.

### 2.2. Weighted Gene Coexpression Network Construction

We created weighted gene coexpression networks using the WGCNA program in order to find coexpression networks and choose genes from various clusters [13]. Initially, all samples and absent genes with an average expression value < 0.5 were grouped together. Second, the “pickSoftThreshold” R-function was used to calculate the ideal soft-threshold power (*β*) in order to more effectively discover strong correlations between modules. Then, using the minimum modulus (200) and merge height (0.9) cutoffs, we carried out a hierarchical clustering analysis to find modules. Then, by analyzing the values of gene significance and module affiliation, we employed the “WGCNA” software package to determine the interaction strengths and the relationship between gene modules and LUAD patients [16]. Finally, we used the “Heatmap” package [17] to obtain different module signature genes according to the first principal component (PC) of module expression and evaluated module–trait relationships based on associations between module signature genes and clinical trait diagnoses. The modules most significantly related to the module–trait relationships were screened for genes contained in the modules.

### 2.3. Differential Gene Expression Analysis

We performed background correction and professional summarization of the gene expression profiling data using the robust multiple array averaging method found in the “affy” package [18] of the R software (Version 4.1.3; https://www.r-project.org/) and the Bioconductor package (http://www.bioconductor.org/). The “DESeq2” tool [19] was used to calculate the differences in gene expression levels between the cancer and control groups. |Log2FC| and *p* adj.<0.01 were the statistical significance criteria used to screen for significant differentially expressed genes (DEGs). DEGs were found in the “ggplot2” package to visualize in the volcano plots [20].

### 2.4. Establishment and Validation of Risk Model

First, we used the “limma” package in R software [21] to find the genes that were differentially expressed (*p* < 0.05) by obtaining the intersection of the genes found in the aforementioned WGCNA with the DEGs, which were then subjected to univariate Cox regression analyses. Subsequently, we identified multiple genes that showed a significant association (*p* < 0.05) with the prognosis of LUAD patients, as determined by the “survival” R package [22]. These genes were then imported into the “glmnet” package [23] for LASSO analysis, which aimed to minimize redundant genes and prevent model overfitting. The gene with the least lambda was then chosen as the target gene for the following stage. Multivariate COX analysis was used to identify the final model genes that would be used to build the prognostic risk model. Next, we used the following algorithm to determine each patient's risk score: the risk score is equal to *Σβi* × Expi, where *β* is the gene's Cox regression coefficient and *i* is its level of expression. Next, the *z*-score was normalized for the risk score. Based on the median risk score in the training cohort, LUAD patients in the validation cohort and training cohort were then split into high-risk and low-risk groups, respectively. Then, using the R software package “survminer,” survival analyses were carried out between the high-risk and low-risk groups. For prognostic analyses, Kaplan–Meier (KM) survival curves were produced, and the log-rank test was used to assess the significance of the differences. Additionally, we computed the area under the curve (AUC) at 1, 2, and 3 years and displayed time-dependent subject work characteristics (receiver operator characteristic (ROC)) curves to evaluate the efficacy of the prediction model using the R package “timeROC” [24].

### 2.5. Immune Infiltration Analysis

The CIBERSORT program in R software [25] was utilized to determine the degree of immune cell infiltration in the dataset samples. The expression data LM22, which contains 22 types of common immune infiltrating cells, was obtained from the official CIBERSORT website (https://cibersortx.stanford.edu/). To determine if immune cell infiltration differed between the two risk groups (*p* < 0.05), a rank-sum test was employed. The Spearman correlation coefficient was used to calculate the relationship between immune cell infiltration and model genes. Three scores make up the ESTIMATE algorithm: StromalScore, ImmuneScore, and ESTIMATEScore. We then used the R software's “ESTIMATE” package to analyze the training set for immune infiltration and expressed the results as corresponding scores. Based on the Spearman correlation coefficient, the model genes' correlation with the StromalScore, ImmuneScore, and ESTIMATEScore scores was computed.

### 2.6. Gene Set Enrichment Analysis

The variations in biological processes and signaling pathways between high- and low-risk groups were examined using the GSEA_4.2.2 program. We performed enrichment analyses on the background gene collection using the “GSVA” package [26], with adj. *p* values < 0.05 being statistically significant. Using the “clusterProfiler” program, assessments of Gene Ontology (GO) and KEGG were conducted [27]. Both the adj. *q*-value and *p* value less than 0.05 were regarded as statistically significant.

### 2.7. Immunotherapy Relevance and Drug Sensitivity Analysis

In order to examine the impact on patients' survival and immunotherapy response, TIDE is a computational technique that characterizes T-cell dysfunction by utilizing the interplay between the tumor's gene expression profiles and the degree of cytotoxic T-lymphocyte infiltration [28]. A low response rate to ICI therapy is indicated by high TIDE scores. Thus, we first evaluated the potential therapeutic benefits of immunotherapy in high- and low-risk groups by assessing the significance of genes for benefiting from immunotherapy using the TIDE (http://tide.dfci.harvard.edu/) database. The “oncPredict” package [29] in the R software was then used to predict the sensitivity of 198 medications from the Genomics of Drug Sensitivity in Cancer (GDSC) database, and the half-maximum inhibitory concentration (IC_50_) values of the medicines for the patients in the training set were determined. Additionally, we determined which medications had a significant association with RiskScore using the Pearson correlation coefficient, classifying a correlation as significant if it was *p* < 0.05 and |cor| > 0.3.

### 2.8. Cell Culture and Transient Transfection

We bought human lung cancer cells (A549) and human lung normal epithelial cells (BEAS-2B) from the Chinese Academy of Sciences' Institute of Biochemistry and Cell Biology. Every cell was cultivated at 37°C in humidified air with 5% CO_2_ in RPMI 1640 medium (Gibco, Grand Island, NY, United States) added with 100 mg/mL streptomycin (Invitrogen, Carlsbad, CA, United States), 10% fetal bovine serum (10% FBS), and 100 U/mL penicillin. The Lipofectamine transfection reagent was applied for the transfection as directed. In detail, Lipofectamine 3000 Transfection Reagent (Thermo Fisher Scientific, Waltham, MA, USA) was applied to transiently transfect si-*ANLN* (anillin actin-binding protein) (sequence: 5⁣′-UUUGUCUCUAGCUUUAUCCTT-3⁣′) into lung cancer cells A549. The cells underwent STR identification, and the results of mycoplasma detection for these cells were found to be negative.

### 2.9. qRT-PCR Analysis

Following the manufacturer's instructions, total RNA was extracted from BEAS-2B and A549 cells with the use of the RNAiso Plus reagent (Takara Bio, Shiga, Japan). To convert RNA to cDNA, PrimeScript RT premix (Takara Bio, Japan) was utilized. Quantification was done using SYBR Green qPCR premix (Vazyme Bio, Nanjing, China). GAPDH served as an internal reference, and SYBR Green qPCR premix (Vazyme Bio, China) was utilized for quantification. Every PCR reaction was conducted in triplicate, and the 2^−ΔΔCT^ technique was used to examine the results.  Table 1 lists the primers for the target genes that were employed in this investigation.

### 2.10. Cell Counting Kit 8 (CCK-8) Assay

Transfected cells (3 × 10^4^ cells/mL) in the logarithmic growth phase were obtained, inoculated, and cultivated in 96-well plates for 48 h. After the cell density reached 80%, the cells were treated for 3 h at 37°C with CCK-8 solution (Dojindo, Tokyo, Japan) (medium: CCK‐8 = 10 : 1), and a microplate reader was used to measure the absorbance at 450 nm. Every experiment was conducted at least three times.

### 2.11. Cell Scratch Assay

An assay for wound healing was used to quantify collective cell movement. Six-well plates were injected with transfected cells (1 × 10^5^ cells/well). A 10 *μ*L plastic pipette tip was used to scratch the monolayer to create a consistent wound after the cell density surpassed 90%. Following a PBS wash, the monolayers were cultured in a medium devoid of FBS. Images were taken at 0 and 48 h to measure the wound edge lengths between the two edges of the migrating cell sheet. Every experiment was carried out three times. Wound closure rate (%) = (Initial wound width − final wound width)/initial wound width × 100%.

### 2.12. Transwell Assay

A transwell chamber (Corning, New York, NY, United States) was used to measure the invasiveness of the cells. Thirty-six hours after cell transfection, 5 × 10^5^ cells were supplemented into the top transwell chamber in 200 *μ*L of serum-free medium, and then 5 × 10^5^ cells were moved to the upper chamber coated with matrix gel (BD Biosciences). Seven hundred microliters of medium containing 10% FBS was filled into the lower chamber, and the incubation process was continued for 48 h. Cells on the filter's lower side were then fixed using 0.1% crystal violet and 4% paraformaldehyde. After that, 4% paraformaldehyde was used to fix the cells on the filter's lower side, and a 0.1% crystal violet solution was used to stain the invaded cells. Finally, six randomly selected fields of view were photographed with a microscope, and the number of invaded cells was quantified.

### 2.13. Statistical Analyses

All statistical analyses for bioinformatics were conducted using R software (v4.2.1). Student's *t*-test or two-way ANOVA followed by Bonferroni's multiple post hoc tests were performed for statistical analyses in GraphPad Prism (v8, GraphPad Software, San Diego, CA, United States). We created KM survival curves and evaluated the variations between the curves. The examination of correlations between continuous variables was done using Spearman's rank correlation. For all analyses, differences were defined significant if *p* value < 0.05.

## 3. Result

### 3.1. WGCNA Screening for LUAD-Related Gene Modules

**Figure 1:**
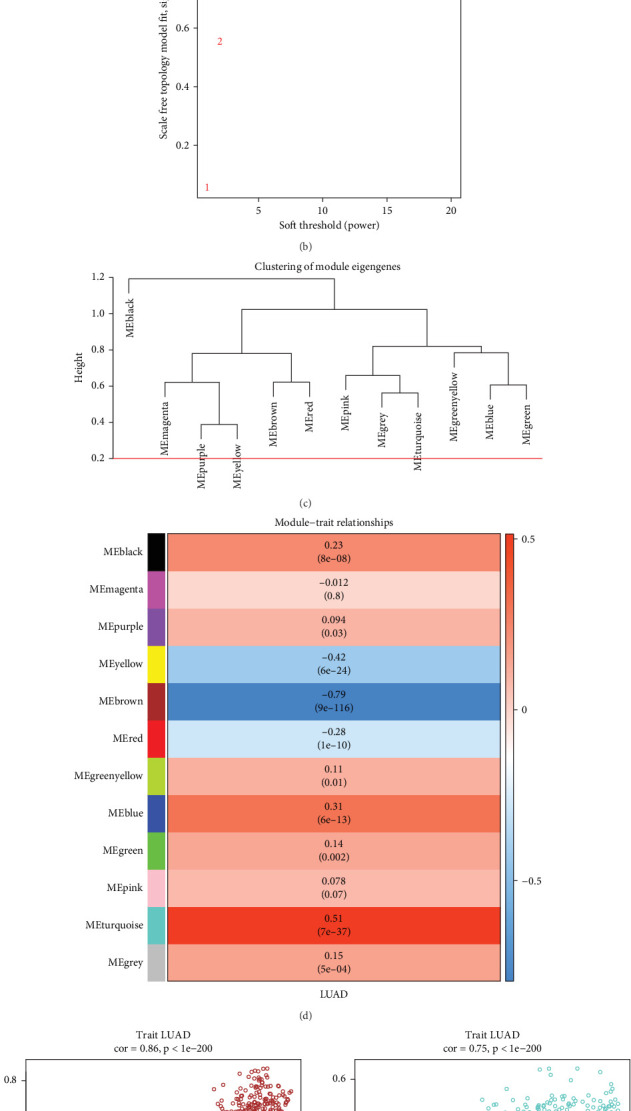
Identification of LUAD-related gene modules in TCGA dataset using WGCNA. (a) Clustering tree of TCGA samples, no obvious outliers were found. (b) Soft threshold screening plot, screening the minimum value of 5 with *R*^2^ higher than 0.90 as the soft threshold for constructing the topological network. (c) Module correlation diagram. (d) Heatmap of module traits for correlations between clustered gene modules and LUAD in TCGA dataset. (e) GS-MM diagram of brown module. (f) GS-MM plot of the turquoise module.

Initially, we created an example clustering tree and ran a WGCNA on 527 samples from TCGA-LUAD dataset. With at least 200 genes per module, we carried out dynamic module identification in several cohorts, and a total of 12 modules were acquired from the division. There were no clear outlier samples discovered, as Figure 1a illustrates. To build the topological network, the soft threshold was then set to 5 (*R*^2^ greater than 0.90). After computing module correlation, it was discovered that there was no module merging (Figure 1b). Then, using the Pearson correlation coefficient to group the samples, we looked at the module correlations with the LUAD samples. The results showed that MEbrown and LUAD had the largest negative association (cor = −0.79, *p* = 9e − 116), whereas MEturquoise and LUAD had the strongest positive connection (cor = 0.51, *p* = 7e − 37, Figure 1c,d). For additional examination, we have determined that the MEbrown and MEturquoise modules are clinically significant. We then examined whether the correlation between MM and GS. In this instance, the MEturquoise module was represented in green, and the MEbrown module was represented in brown. We found a strong positive correlation between MM and GS in the brown and green modules (cor = 0.86 and cor = 0.75, Figure 1e,f).

### 3.2. Screening of DEGs in LUAD Samples and Paracancerous Samples

**Figure 2:**
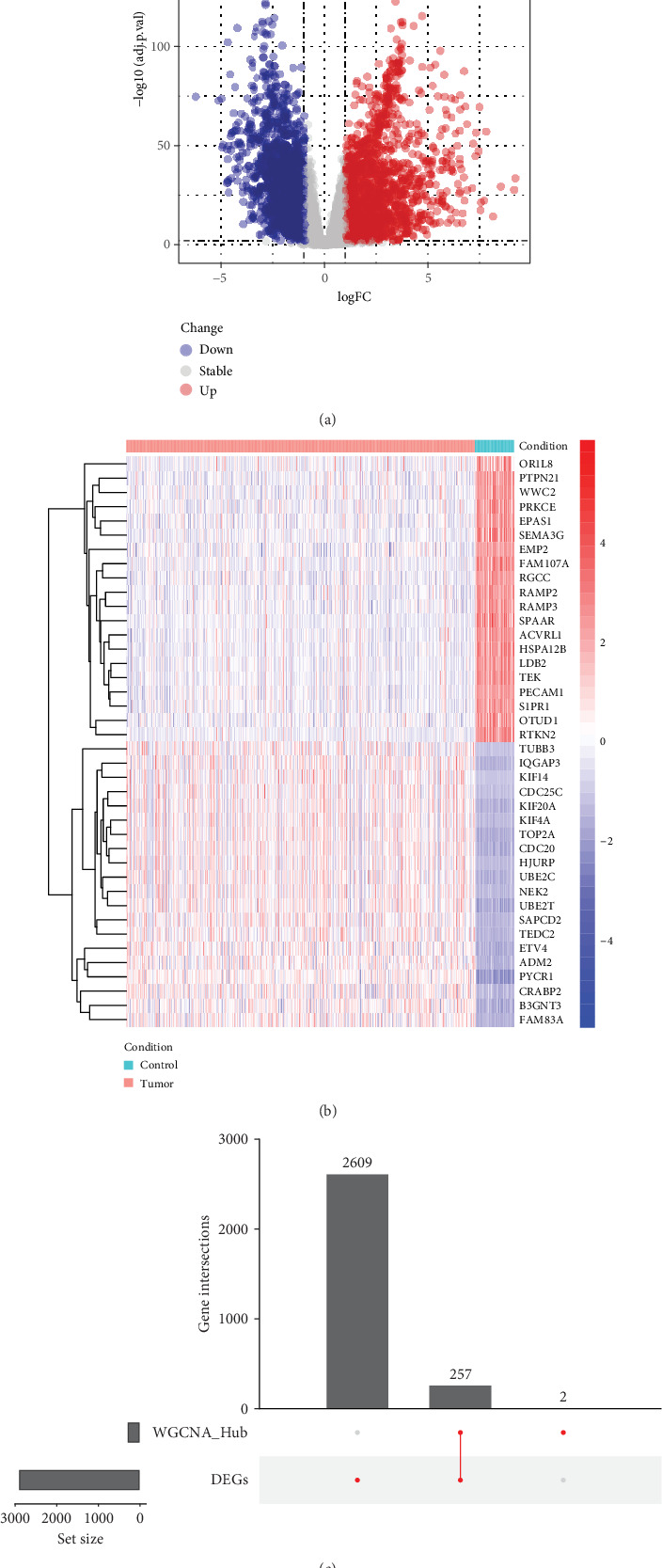
DEGs of 527 samples from the screened TCGA dataset. (a) The volcano plots displaying the DEGs. (b) Heatmap of gene expression. (c) WGCNA versus differentially expressed gene upsets plot.

We next used the “DESeq2” tool to assess the changes in gene expression levels between tumor samples and paracancerous samples. Using |log2FC| > 1 and *p* adj.<0.01 as the criteria, we screened for significant DEGs. In the end, 2866 differently expressed genes were found, comprising 1390 upregulated genes and 1476 downregulated genes.  Figure 2 displays the volcano map displaying the DEGs. A negative association was seen between the up- and downregulated DEGs in tumor samples and paracancerous samples, according to the gene expression heatmap created by selecting the Top 20 significantly upregulated DEGs and the Top 20 significantly downregulated DEGs (Figure 2). Finally, we took all the genes within the two modules obtained from WGCNA screening and the intersecting genes of the DEGs between the tumor samples and the paracancer samples, and the final 257 intersecting genes obtained were used for the subsequent analysis (Figure 2).

### 3.3. Prognostic Modeling and Validation

**Figure 3:**
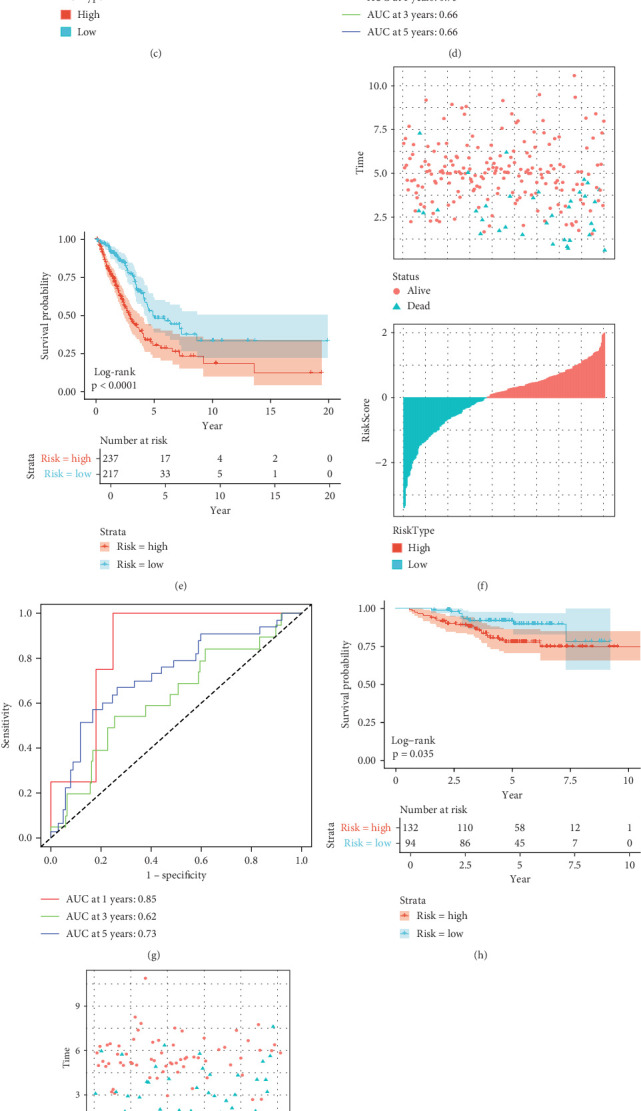
Risk model construction and validation based on intersecting genes. (a) Plot of parameters of LASSO regression analysis with log(lambda) values in horizontal coordinates and degrees of freedom in vertical coordinates, representing the error of cross-validation. (b) Plot of LASSO regression coefficients. (c–k) Survival time, survival analysis of the Kaplan–Meier curves, and ROC curves for patients in TCGA-LUAD cohort, GSE31210 cohort, and GSE50081 cohort.

Here, we performed a unvariate Cox analysis on the 257 intersecting genes that we had acquired earlier, and the results showed that 176 genes were substantially related to the survival in patients with LUAD. Nevertheless, a significant percentage of these genes also hurt clinical testing. We further reduced the number of these 176 prognostically relevant genes using LASSO regression. After determining that the model was at its best at lambda = 0.067, we chose the genes *ANLN*, *CASS4* (Cas scaffolding protein family member 4), and *NMUR1* (neuromedin U receptor 1) as our target genes for the following stages (Figure 3). The risk score for every patient in TCGA cohort was then determined using the following formula, which we derived using the coefficients of these genes: risk score = 0.26∗*ANLN* + −0.266∗*CASS*4+−0.574∗*NMUR*1.

Each sample's risk score was determined using the risk model's methodology, and the risk score was then *z*-score standardized. Based on the threshold value of “0,” the samples in TCGA-LUAD training cohort were split into high-risk and low-risk groups. The patients' overall survival (OS) of the two groups was then contrasted. According to the findings, patients in the low-risk group outlived those in the high-risk group in terms of OS (*p* < 0.0001, Figure 3). We then looked at how long LUAD patients survived across various subgroups and discovered that, on average, the survival time of high-risk patients was shorter than those with a low risk (Figure 3). Additionally, we used the R software package's “timeROC” function to analyze the risk score prognostic classification's time-dependent ROC curve. The validity of the prognostic prediction classification at 1, 3, and 5 years was examined individually. The AUC values for 1, 3, and 5 years were 0.73, 0.66, and 0.66, respectively (Figure 3). These results demonstrate the model's good prediction ability for the prognosis of LUAD patients.

We employed the cohorts of training set GSE31210 and validation set GSE50081 to evaluate the robustness of the models using models and equivalency coefficients similar to those used for the identified training set. As can be seen, GSE31210 showed similar results (*p* < 0.035, Figure 3) and GSE50081 validation set cohorts (*p* < 0.00018, Figure 3), with a significantly lower OS and survival time in the high-risk group of patients with LUAD as compared to the low-risk group (Figure 3, 3, 3, and 3) GSE31210 validation set. The AUC values of 1, 3, and 5 years in the cohort were as high as 0.85, 0.62, and 0.73, respectively (Figure 3). The AUC values of 1, 3, and 5 years in the GSE50081 validation set cohort were 0.71, 0.69, and 0.65, respectively (Figure 3). It indicates that the model genes are good prognostic predictors for LUAD patients.

### 3.4. Relationship Between Independent Prognosis and Clinicopathological Characteristics of LUAD Patients

**Figure 4:**
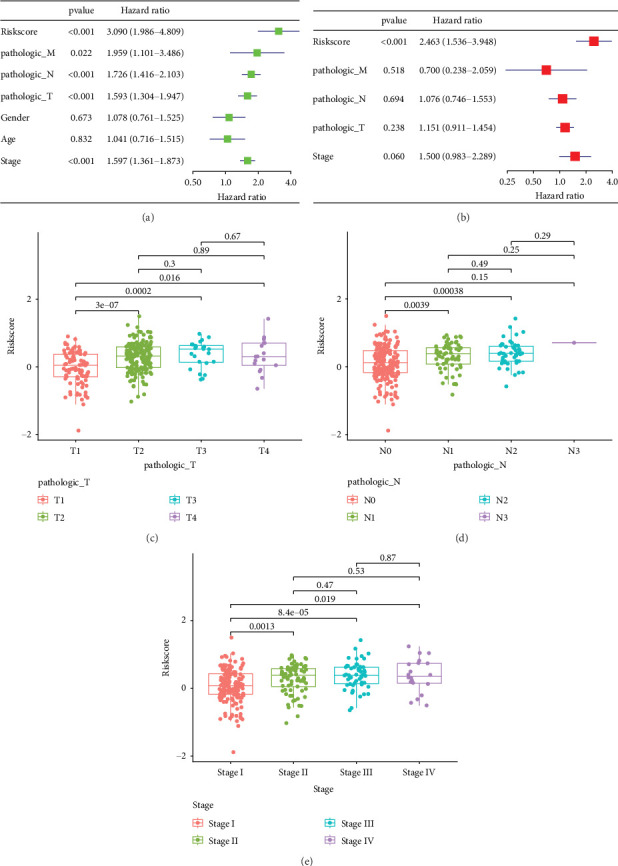
Distribution of risk scores for different clinicopathological features in LUAD patients. (a, b) Univariate and multivariate Cox analyses of common clinical parameters and Riskscore. (c–e) Box line plots of the relationship between Riskscore and pathologic_T, pathologic_N, and Stage.

We conducted univariate and multivariate Cox regression analysis on TCGA-LUAD training set to further ascertain whether risk score is an independent clinical prognostic factor independent of other covariates. Initially, we used clinical information from TCGA-LUAD patient group. Risk score, pathologic_T, pathologic_N, pathologic_M, and tumor stage were risk variables affecting OS in LUAD patients, according to univariate Cox analysis (Figure 4). After correcting for clinicopathological variables, we then verified using multifactorial Cox regression analysis that risk score was an independent risk factor influencing OS in LUAD patients (Figure 4). We examined the variations in risk score between clinicopathological features in TCGA-LUAD cohort in order to better understand the link between risk score and these features. The results of the study showed significant differences in risk score between different pathologic_T, pathologic_N, and tumor stage, indicating that risk score has potential prognostic predictive value (Figures 4, 4, and 4).

### 3.5. Analysis of Immune Infiltration in Patients With LUAD

**Figure 5:**
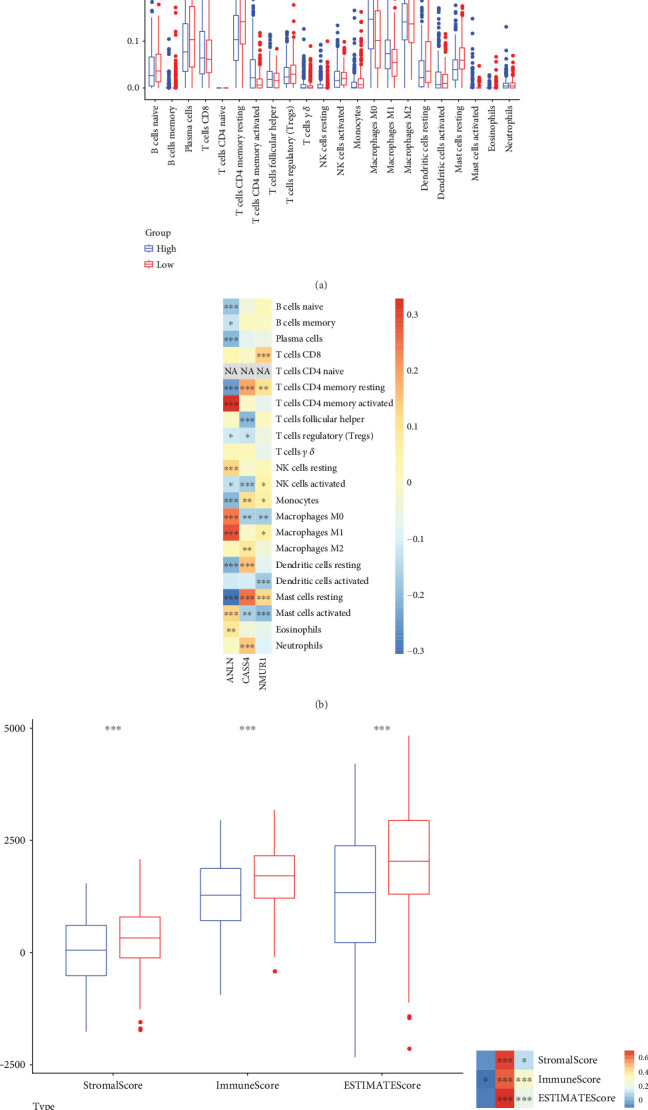
Immunological characteristics between high- and low-risk subgroups of LUAD patients. (a) Box line plot on the infiltration of 22 types of immune cells based on CIBERSORT. (b) Spearman's correlation analysis of three biomarkers with 22 immune cell infiltration profiles. (c) ESTIMATE assessment of differences in immune infiltration scores between high- and low-risk subgroups. (d) Spearman's correlation analysis of three biomarkers with immune infiltration status. *ns* (*p* >0.05): not statistically significant; ⁣^∗^*p* < 0.05; ⁣^∗∗^*p* < 0.01; ⁣^∗∗∗^*p* < 0.001.

We used CIBERSORT to analyze the immune cell infiltration of 474 tumor samples between high- and low-risk groups of LUAD patients. The results showed higher levels of infiltration of some immune cells in the low-risk group relative to patients in the high-risk group, for example, plasma cells, T cell CD4 memory resting, T cell memory activated, and mast cells resting, among others (Figure 5a). The expression of model genes was related to the infiltration of the great majority of immune cells, with the correlation between *ANLN* genes and immune cell infiltration being generally strong. We then computed the relationship between model gene expression profiles and immune cell infiltration scores (Figure 5b). We evaluated the degree of immune cell infiltration in LUAD patients in order to further detect variations in the patient's immunological milieu between high- and low-risk groupings. The findings demonstrated that the low-risk group had significantly higher levels of immune cell infiltration than the high-risk group, as indicated by the stromal infiltration score, immunity score, and ESTIMATE score (Figure 5c). The expressions of model genes *NMUR1* and *CASS4* were closely related to the stromal infiltration score, immunity score, and ESTIMATE score, while the immunity score showed a significant correlation with the *ANLN* gene (Figure 5d). These findings are consistent with our analysis of differences in model gene expression and immune microenvironment.

### 3.6. GSEA of Differences in Marker Gene Sets Between Patients in Different Risk Groups for LUAD

**Figure 6:**
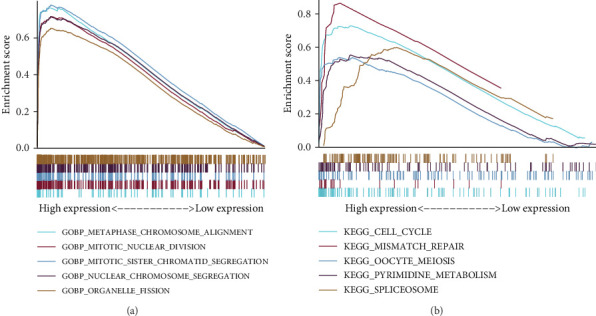
Functional enrichment analysis between patients in different LUAD risk groups. (a) GSEA of GO results between high- and low-risk groups. (b) GSEA of KEGG results between high- and low-risk groups.

In order to investigate the manner in which risk score impacts LUAD patients, we looked at the variations in biological processes and signaling pathways across high- and low-risk groupings. Compared with the low-risk group, the biological functions of patients in the high-risk group were primarily enriched in METAPHASE_CHROMOSOME_ALIGNMENT, MITOTC_NUCLEAR_DIVISION, MITOTC SISTER_CHROMATID_SEGREGATION, NUCLEAR_CHROMOSOME_SEGREGATION, and ORGANELLE_FISSION responses, according to enrichment analysis in the background gene set using GSEA software (FDR < 0.05, Figure 6). Similar to this, patients in the high-risk group exhibited high levels of activity in the signaling pathways CELL_CYCLE, MISMATCH_REPAIR, OOCYTE_MEIOSIS, PYRIMIDINE_METABOLISM, and SPLICEOSOME (Figure 6). This suggests that cell division and related pathways, such as the cell cycle, played a major role in different biological functions between patients in the high- and low-risk groups.

### 3.7. Immunotherapy and Drug Sensitivity Assessment of Patients in Different LUAD Risk Groups

**Figure 7:**
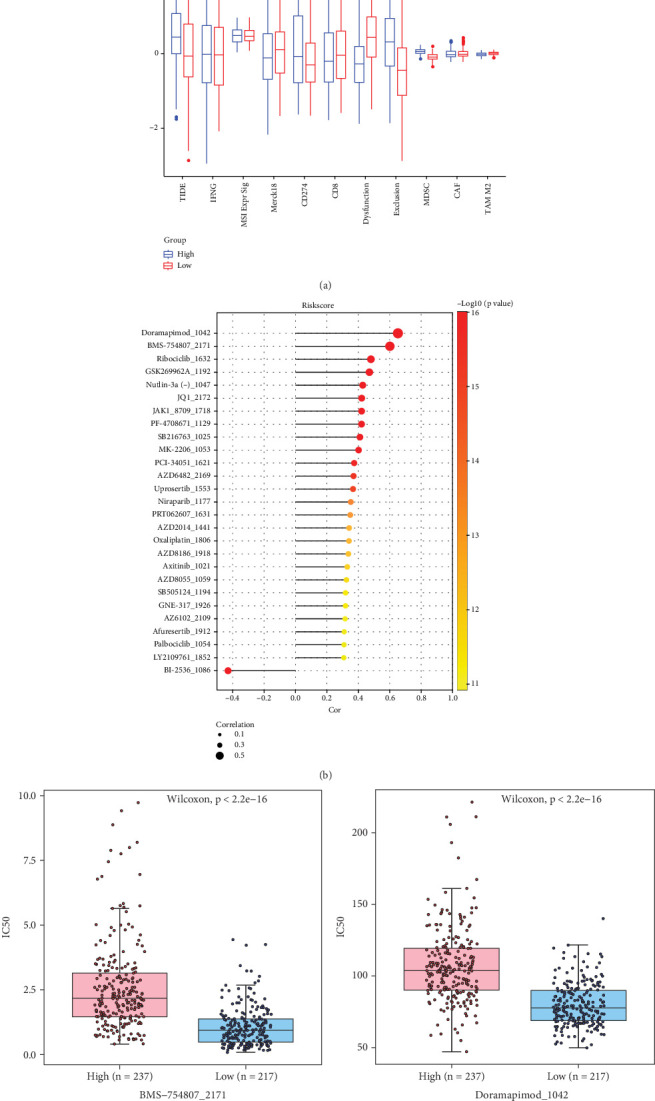
Risk model with immunotherapy and drug sensitivity differences between patients in different LUAD risk groups. (a) TIDE software predicts immunotherapy marker scores between risk subgroups. (b) Lollipop plot of correlation between Riskscore and sensitivity to 27 drugs. (c) IC_50_ boxplot of BMS-754807_2171 across risk subgroups. (d) IC_50_ box line plot of Doramapimod_1042 in different risk subgroups. *ns* (*p* >0.05): not statistically significant; ⁣^∗^*p* < 0.05; ⁣^∗∗^*p* < 0.01; ⁣^∗∗∗^*p* < 0.001.

The possible clinical effects of immunotherapy between high- and low-risk groups were then evaluated using TIDE software. The seven immunotherapy marker scores varied considerably between the risk groups, as Figure 7a illustrates. The low-risk group's higher scores for tumor inflammatory profile (Merck18), dysfunction, and M2 subtype tumor-associated macrophages (TAM). In the high-risk group, the greatest scores were obtained by TIDE, CD274, myeloid-derived suppressor cells (MDSCs), and exclusion. This implies that immunological rejection processes may be the main means by which patients in the high-risk category avoid the immune system. We next conducted a correlation analysis between Riskscore and 198 drug sensitivities from the GDSC2 database in an effort to find medications with substantial differences in order to clarify the relationship between the model and drug sensitivities. According to calculations, there was a substantial correlation between Riskscore and 27 medication sensitivities (Figure 7b). With the exception of medication BI_2536_1086's sensitivity, which exhibited a negative association with Riskscore, all 26 drug sensitivities among them demonstrated a positive correlation with Riskscore. Furthermore, the chemotherapeutic medicines' responsiveness in both groups of LUAD patients should be evaluated. Based on the risk group, we chose BMS-754807_2171 and Doramapimod_1042—the two medications with the highest correlation—for sensitivity testing. These results indicated that high-risk patients could benefit less from treatment. Specifically, high-risk LUAD patients exhibited a greater IC_50_ (*p* = 2.2e − 16) to the medications BMS-754807_2171 versus Doramapimod_10424 (Figure 7c,d).

### 3.8. Knocking Down ANLN Remarkably Suppressed the Proliferation, Migration, and Invasion of LUAD Cell Line A549

**Figure 8:**
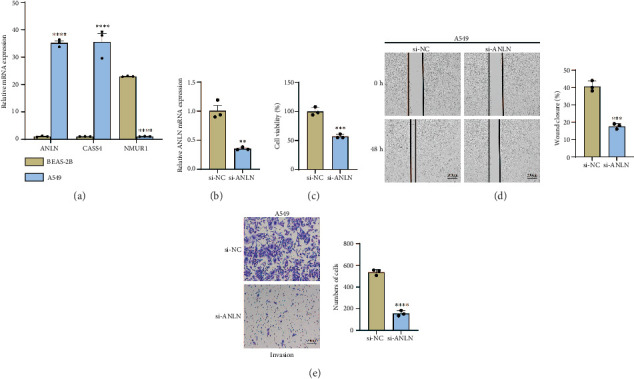
Exploring the biological role of *ANLN* in LUAD. (a) qPCR detection of the expression levels of *ANLN*, *CASS4*, and *NMUR1* in BEAS-2B cells and A549 cells. (b) qRT-PCR-based to validate the knockdown efficiency of *ANLN*. (c) Verification of the effect of si-*ANLN* on A549 cell viability. (d) Representative images and statistical analysis of wound healing assay in A549 cells after *ANLN* knockdown. (e) Representative images of transwell assay of A549 cells after *ANLN* knockdown and statistical analysis of invasive cell counts. Data are expressed as SD ± mean; ns (*p* >0.05): not statistically significant; ⁣^∗∗^*p* < 0.01; ⁣^∗∗∗^*p* < 0.001; ⁣^∗∗∗∗^*p* < 0.0001.

According to qRT-PCR results, *ANLN* and *CASS4* gene levels were considerably higher in A549 cells than in BEAS-2B cells. Additionally, A549 cells had substantially less *NMUR1* mRNA expression than BEAS-2B cells (Figure 8). *ANLN* was related to the poor prognosis, lymph node metastasis, distant metastasis, tumor size, and degree of differentiation in colorectal cancer [30] and pancreatic cancer [31]. As shown in Figure 8, the PCR results showed that silencing *ANLN* resulted in a significant decrease in the *ANLN* expression level in A549 cells, which indicated the successful knockdown of *ANLN*. Here, we validated the potential role of *ANLN* by knocking it down to the LUAD cell line. Based on the data of CCK-8 assay, we found that *ANLN* knockdown significantly reduced the viability of A549 cells 48 h later (Figure 8). It was discovered that the incidence of metastasis and invasion of tumor cells was linked to the poor prognosis of LUAD patients [32]. Wound healing assay and transwell assay demonstrated that the migration and metastasis of A549 cells were markedly suppressed by the lowering of *ANLN* (Figure 8).

## 4. Discussion

Over the past 10 years, advances in LUAD research have been made by humans, and targeted and immunotherapeutic medications are continuously being developed. Lung cancer treatment is becoming more promising thanks to novel therapeutic agents and multidisciplinary treatment approaches [33]. But there has not been much of an improvement in lung cancer survival rates, and finding new, reliable markers to inform clinical treatment choices is still a requirement. Machine learning algorithms and WGCNA have advanced with the diversification of informatics technologies and are now frequently utilized for the prediction of therapeutic targets and disease indicators in a range of tumor types [34]. Based on machine learning algorithms and WGCNA, researchers have currently performed predictive studies of prognosis and biomarkers in patients with ovarian and gastric malignancies, among other cancers [35, 36]. Important genes linked to immune evasion and the epithelial–mesenchymal transition in LUAD were found by researchers using WGCNA, and these genes were linked to worse patient outcomes, altered tumor microenvironment (TME), decreased treatment sensitivity, and a higher frequency of mutations [37]. Cuproptosis-related genes have been found to positively correlate with OS in prognostic models, and variations in immune cell infiltration and cancer treatment susceptibility have been found in patients with LUAD [38]. Using four differentially expressed optimum disulfidptosis/ferroptosis enrichment-related genes related to the prognosis in LUAD patients by WGCNA and unsupervised clustering, Ma et al. predicted the outcome of immunotherapy in LUAD patients [39]. Using a combination of standard bioinformatics techniques, we were able to identify three hub genes associated with prognosis from the prognosis-related genes of LUAD patients: *ANLN*, *CASS4*, and *NMUR1*. The Riskscore based on the three hub genes displayed good diagnostic efficacy for OS in LUAD patients, according to the results of the ROC curve study.

*ANLN*, *CASS4*, and *NMUR1* are three prognosis-related genes for which we generated a risk score, which is noteworthy in comparison to the prognostic models built by previous researchers [40]. This risk score may be highly useful for clinical application. One gene that codes for proteins is the *ANLN*. Previous research has demonstrated that ANLN knockdown activates the pathway linked to pyroptosis and that elevated *ANLN* expression in LUAD is linked to a bad prognosis for patients [41]. In addition, a growing number of studies have found that targeting *ANLN* in the ceRNA regulatory network suppresses antitumor immunity in the tumor immune microenvironment and enhances multidrug resistance in tumors [42]. As a result, *ANLN* has the potential to function as a cancer immune biomarker and can be employed in tumor screening, prognosis, tailored therapy planning, and post-treatment monitoring [43]. We also found high expression of *ANLN* mRNA in A549 cells, which significantly promoted proliferation, migration, and invasion of A549 cells. Furthermore, it has been discovered that overexpressed *CASS4* stimulates the AKT signaling pathway and inhibits E-calmodulin, hence promoting NSCLC invasion [44]. We also found that *NMUR1* mRNA was highly expressed in A549 cells. Upregulation of *CASS4* in LUAD was linked to a poor prognosis for patients, according to Zhao et al. [45]. *NMUR1* was discovered to be extensively dispersed throughout the body's organs and was linked to a poor prognosis in individuals suffering from squamous cell carcinoma of the head and neck [46]. A previous study reported that downregulation of *NMUR1* in LUAD negatively affected OS, which may be a favorable prognostic factor [47]. This is consistent with our findings that *NMUR1* mRNA expression is low in A549 cells. ssGSEA and CIBERSORT analyses also found *NMUR1* to be negatively associated with LUAD immune cell infiltration and immunotherapy [48]. In accordance with the available analyses, these three hub genes are mainly associated with cell invasion, metastasis, immune infiltration, and response to therapy, which may be of potential value in prognostic prediction in LUAD.

In addition, epidemiological studies have shown that lower immune scores, higher tumor purity, and significantly lower abundance of most immune cells and immune-related features of the immune microenvironment are associated with poorer OS in patients with LUAD [49]. Patients at high risk of LUAD had higher tumor purity, lower immunological scores, and considerably lower abundance of most immune cells and immune-related characteristics, according to research by Ma et al. and Jayadev and Yusuff [49, 50]. For this reason, investigating the relationship between prognosis and tumor immune function is helpful in the identification and management of LUAD. According to the current characterization, immunological infiltration of T cells_CD4_memory_activated, macrophages_M0, and macrophages_M1 in high-risk patients was positively related to the risk score. The immune infiltration of mast cells, dendritic cells, T cells, CD4 memory resting, and plasma cells was higher in patients in the low-risk group. Previous studies have found that macrophage and T-cell CD4 memory activation in the LUAD immunological landscape is relatively upregulated in patients with LUAD TP53 mutations, potentially playing a role in the suppression of cancer metastasis and the benefit of patient immunotherapy [51]. According to Hao et al. and Foote et al., there was a strong positive association between a higher OS among patients and the presence of highly concentrated plasma cells in LUAD tumor tissues [9, 52]. According to Koh et al., they noted that the infiltration abundance of T_cells_CD4_memory_resting favored immunotherapy in LUAD patients, whereas the number of mast cells infiltrated promoted immunosuppressive status in LUAD patients [53]. In conclusion, the immune status of patients is indicated by the infiltration of immune cells in LUAD, and this could be the reason for the disparity in survival rates between the two risk groups.

GO and KEGG pathways between the two risk groups were analyzed using GSEA in order to reveal the mechanism of the identified genes. Chromosome division, mitosis, and organelle division are considered to be the most active biological processes in the high-risk group. These roles are very important in determining the prognosis of LUAD patients and are believed to be directly related to cell proliferation, apoptosis, migration and invasion, N and M types, and clinical staging [54, 55]. Similarly, signaling pathways such as spliceosome, cell cycle, and cell division were most significantly enriched, mainly in the high-risk group. These pathways are thought to be associated with poor prognosis, cycle blockade, immune infiltration, and suppression of immunoreactivity in LUAD [56, 57]. The aforementioned findings imply that the poor prognosis of the LUAD high-risk group may be mostly due to the interplay of these pathways with the three essential genes.

One major factor contributing to tumor therapy's ineffectiveness is immune escape. The capacity of the tumor to deliver antigens, Merck18, immune checkpoint expression, and tumor immunogenicity are the primary immune escape strategies from tumors, according to studies on the subject [58]. Even though certain TMEs exhibit substantial cytotoxic T-cell infiltration, these T cells are typically toward the end of their functional life and no longer have the capacity to eradicate tumors. However, T cell penetration into the TME is restricted by the existence of immunosuppressive cells (MAF, MDSC, and TAM) [59]. Nevertheless, effective tumor immunosuppression is not achieved by inhibiting PD-1/CD274 (PD-L1) in conjunction with T cell reduction and/or malfunction in TME [60]. Jiang et al. developed an algorithmic framework for TIDE based on the genomic characterization of T-cell dysfunction and T-cell rejection [60], which can calculate the T-cell dysfunction in TME and the T-cell exclusion scores as well as TAM, CAF, and MDSC infiltration levels. Of note, we employed the TIDE software to examine the possible clinical effects of immunotherapy between the two risk groups of LUAD and discovered that TIDE, CD274, MDSC, and exclusion had the highest scores in the high-risk group. From this, it was seen that high-risk patients may evade the immune system predominantly through the process of immunological rejection. However, the low-risk group of patients may rely predominantly on immune cell malfunction for immunological escape, as shown by the higher scores of TAM, dysfunction, and Merck18 in that group. Furthermore, prior research has demonstrated a negative correlation between DEGs and barrier scores, Merck18, and a positive correlation with MDSC in LUAD, which is in line with our findings [61, 62]. The survival rate of LUAD patients is significantly reduced by the frequent onset of treatment resistance, which significantly affects the therapeutic decisions made by physicians [63]. As a result, a thorough examination of LUAD patients' drug sensitivity is required. In this investigation, we ran a correlation analysis using the GDSC2 database's 198 medication sensitivities and Riskscore. Medicines BMS-754807_2171 and Doramapimod_1042 were found to have the strongest link with Riskscore. Additionally, high-risk LUAD patients exhibited greater IC_50_ for these medicines, which could perhaps account for the high-risk group's poor prognosis. According to earlier research, BMS-754807 enhanced the effects of chemotherapeutic drugs on lung cancer cells by causing autophagy, cycle arrest, and growth inhibition, all of which resulted in synergistic cytotoxicity [64, 65]. Furthermore, doramapimod promotes apoptosis and suppresses proliferation in non–small cell lung cancer cells [66]. In conclusion, our research may offer some theoretical groundwork for LUAD patients to receive individualized care.

There are certain limits, despite the fact that our model performed admirably in both the training and validation cohorts. Initially, our findings are derived from a restricted patient population in a public database. Secondly, the patient pool was assembled after the fact, which could introduce bias and necessitate validation by prospective research. In addition, further experimental studies and clinical validation of the biological functions of the hub genes in our model are lacking. Additionally, these genes' biological roles are crucial for future experiments. Consequently, more multicenter randomized controlled studies with excellent quality, sizable sample sizes, and sufficient follow-up data are needed for verification.

## 5. Conclusion

In this study, we used machine learning methods and screened two module genes with the strongest correlation with LUAD and then took the intersection of them with the genes related to the prognosis of LUAD and screened three feature genes to construct a prognostic model, which has strong robustness. It can stably predict the prognosis, immune infiltration level, biological pathways, immunotherapy, and sensitivity to anticancer drugs in LUAD patients, offering a reliable basis for personalized treatment. This study may provide a theoretical reference for future exploration of potential diagnostic and prognostic biomarkers for LUAD patients.

## Figures and Tables

**Table 1:** The sequences of primers for RT-qPCR used in this study.

**Gene**	**Sequence**
ANLN	F: CAGACAGTTCCATCCAAGGGAG
	R: CTTGACAACGCTCTCCAAAGCG
CASS4	F: GACTCTGCCTTCCCAGGTGTAT
	R: GGATGGAGTCCTGCCTTCTTGG
NMUR1	F: ACAGCGACTATCACAGAGTCCG
	R: GCCCAGCAGA-TCCCAAACAC
GAPDH	F: GTCTCCTCTGACTTCAACAGCG
	R: ACCACCCTGTTGCTGTAGCCAA

## Data Availability

The datasets generated and/or analyzed during the current study are available in the GSE31210 repository (https://www.ncbi.nlm.nih.gov/geo/query/acc.cgi?acc= GSE31210) and the GSE50081 repository (https://www.ncbi.nlm.nih.gov/geo/query/acc.cgi?acc= GSE50081).

## Nomenclature

LUAD: lung adenocarcinoma
ICB: immune checkpoint blockade
WGCNA: weighted gene coexpression network analysis
GS: gene significance
MM: module affiliation
KEGG: Kyoto Encyclopedia of Genes and Genomes
TAM: M2 subtype tumor-associated macrophage
MDSCs: myeloid-derived suppressor cells
Merck18: tumor inflammatory profile
KM: Kaplan–Meier
GDSC: Genomics Drug Sensitivity in Cancer
IC_50_: inhibitory concentration
AUC: area under the ROC curve
DCA: decision curve analysis
GEO: Gene Expression Omnibus
LASSO: least absolute shrinkage and selection operator
OS: overall survival
ROC: receiver operating characteristic analysis
GSVA: gene set variant analysis
ssGSEA: single-sample gene set enrichment analysis
TCGA: The Cancer Genome Atlas
TIDE: tumor immune dysfunction and exclusion
TME: tumor microenvironment
ICIs: immune checkpoint inhibitors
